# Hypoxia disrupts neurovascular regulation of blood pressure in normotensive and untreated hypertensive men

**DOI:** 10.1007/s10286-025-01135-7

**Published:** 2025-06-18

**Authors:** Qudus A. Ojikutu, Jeann L. Sabino-Carvalho, Katherine Latham, Marcos Rocha, Joao D. Mattos, Monique O. Campos, Daniel E. Mansur, Lauro C. Vianna, Antonio C. L. Nóbrega, Igor A Fernandes

**Affiliations:** 1 Human Neurovascular Control Laboratory, Department of Health and Kinesiology, Purdue University, West Lafayette, IN USA; 2 Division of Renal Medicine, Department of Medicine, Emory University School of Medicine, Atlanta, GA USA; 3 August Krogh Section for Human and Molecular Physiology, University of Copenhagen, Copenhagen, Denmark; 4 Laboratory of Exercise Sciences, Department of Physiology and Pharmacology, Fluminense Federal University, Niterói, Rio de Janeiro Brazil; 5 Indiana University School of Medicine, Indianapolis, IN USA; 6 NeuroV̇ASQ̇, Integrative Physiology Laboratory, Faculty of Physical Education, University of Brasília, Brasília, Federal District Brazil

**Keywords:** Hypertension, Sympathetic nervous system, Arterial baroreflex

## Abstract

**Background:**

Hypoxia is a common feature of arterial hypertension that does not consistently elevate blood pressure (BP), but triggers exaggerated increases in muscle sympathetic nerve activity (MSNA) and may disturb sympathetic transduction and baroreflex sensitivity in hypertensive individuals. Elevated resting MSNA, enhanced sympathetic transduction, and reduced baroreflex sensitivity are all associated with increased blood pressure variability (BPV), a marker of target organ damage independent of absolute BP levels. We hypothesized that hypoxia would elicit greater BPV in hypertensive individuals compared to normotensive controls

**Methods:**

Nine young- to middle-aged men with untreated stage 1–2 hypertension (HT) and normotensive controls (NT) were exposed to normoxia (21% O_2_) and isocapnic hypoxia (IH, 10% O_2_). During both conditions, oxygen saturation, beat-to-beat BP, MSNA, and end-tidal CO_2_ (PetCO_2_) were continuously monitored, with PetCO_2_ clamped. BPV was quantified using standard deviation, coefficient of variation, and average real variability for systolic (SBP), diastolic (DBP), and mean BP (MBP). Sympathetic transduction was assessed using a time-domain signal averaging technique. Cardiac baroreflex sensitivity (cBRS) was evaluated using the sequence method, and sympathetic baroreflex sensitivity (sBRS) was calculated via MSNA–DBP regression

**Results:**

IH induced comparable oxygen desaturation in both groups (NT: −25.7 ± 3.3% vs. HT: −21.2 ± 4.0%, *p* > 0.05). Although BP and PetCO_2_ remained unchanged, MSNA responses were significantly greater in HT (NT: +8 ± 2 vs. HT: +12 ± 2 bursts/min, *p* = 0.03). IH increased all indices of BPV and sympathetic transduction, while both cBRS and sBRS were similarly impaired in the two groups.

**Conclusions:**

In conclusion, IH similarly exacerbates BPV and disrupts sympathetic transduction and baroreflex function in normotensive and untreated hypertensive men, despite greater MSNA reactivity in the hypertensive group.

## Introduction

Epidemiological and mechanistic evidence strongly links acute apneic episodes—such as those caused by the intermittent collapse of the pharynx during sleep—to the onset and progression of cardiovascular disease [1]. These episodes result in abrupt decreases in arterial oxygen levels (hypoxia), often accompanied by unchanged or elevated carbon dioxide levels (hypercapnia). This combination triggers sympathoexcitation via carotid and/or central chemoreflex activation [2]. The resulting fluctuations in arterial blood gases are hypothesized to cause transient spikes in nocturnal blood pressure. Over time, the repetitive nature of these apneic episodes and the associated hemodynamic instability likely contribute to the development of sustained hypertension during waking hours [1, 2]. Notably, acute hypoxemic episodes are highly prevalent among individuals with essential hypertension [2, 3]. Although hypertension is associated with heightened carotid body sensitivity and consequent exaggerated sympathoexcitation during acute hypoxic episodes, hypertensive individuals do not exhibit an increase in blood pressure under hypoxic conditions [4, 5]. In hypertensive individuals experiencing moderate to severe nocturnal hypoxemic episodes, a non-dipping blood pressure pattern (defined as a < 10% decline during nighttime) has also been observed more frequently than hypertension itself [6].

A possible explanation for this blunted pressor response is impaired vascular transduction—the process by which sympathetic nerve activity is translated into vascular tone. Previous studies have shown that acute hypoxia can attenuate sympathetic transduction in young normotensive individuals [7], supporting the notion that altered vascular transduction contributes to the dissociation between sympathetic activation and blood pressure regulation. However, other findings indicate that the ability of sympathetic activity to mediate vasoconstriction may be enhanced during brief exposures to isocapnic hypoxia, counteracting the potent vasodilatory stimulus of hypoxia [8]. This highlights the dynamic and context-dependent nature of sympathetic neurovascular transduction under hypoxic conditions. Notably, sympathetic transduction seems to be impaired in untreated hypertensive individuals, suggesting that higher levels of muscle sympathetic nerve activity (MSNA) are required to achieve comparable vasoconstrictor responses [9]. Whether the uncoupling between exaggerated sympathetic activation and the blunted pressor response during hypoxia in hypertensive individuals reflects further impairment in sympathetic transduction remains to be determined.

While the acute blood pressure response may not fully capture the heightened cardiovascular risk in hypertensive individuals exposed to hypoxia, emerging evidence suggests that blood pressure variability (BPV), the beat-to-beat fluctuations in blood pressure, may serve as a more accurate predictor of cardiovascular risk and end-organ damage than blood pressure alone [10, 11]. Given that alpha-adrenergic receptors are involved in BPV regulation [12] and that elevated BPV is strongly associated with increased sympathetic nerve activity [13], it is plausible that hypertensive individuals experience a more significant disturbance in BPV during hypoxia due to exaggerated sympathoexcitation and disturbed sympathetic transduction. A hypothetical hypoxia-induced impairment in baroreflex sensitivity [14] would also further destabilize blood pressure control, contributing to significant disturbance of BPV under hypertensive conditions. Nonetheless, the effects of acute hypoxia on sympathetic transduction, BPV, and baroreflex sensitivity in hypertensive individuals have yet to be investigated.

This study aims to address this gap by investigating the effects of acute isocapnic hypoxia on sympathetic transduction, BPV, and baroreflex sensitivity in young- to middle-aged hypertensive men. We hypothesize that, in addition to the exaggerated rise in MSNA, hypertensive individuals will exhibit more pronounced disturbances in sympathetic transduction, arterial baroreflex sensitivity and BPV in response to hypoxia compared to their normotensive counterparts. By uncovering these underlying mechanisms, this study aims to advance our understanding of the cardiovascular risks associated with hypoxia in individuals with hypertension.

## Methods

The experimental procedures were approved by the Ethical Committee for Research of the Fluminense Federal University (CAAE: 54362116.3.0000.5243) following the Declaration of Helsinki, except for registration in a database. Informed consent was obtained from each participant prior to their involvement in the study. Before engagement, all subjects received a thorough explanation of the experimental procedures, had the opportunity to review the protocol in detail, and were encouraged to ask questions.

The primary focus of the study was to investigate BPV, sympathetic transduction, cardiac, and sympathetic arterial baroreflex responses to hypoxia in hypertensive individuals. Notably, a subset of the data presented in this manuscript—BP, heart rate (HR), ventilation $(\dot{\text {V}}\text {E})$, and MSNA burst frequency (BF) and incidence (BI)—was previously published as part of a study addressing different research questions [4].

### Participants

We enrolled 18 male participants, including nine individuals with untreated stage 1–2 hypertension (HT group, aged 44 ± 12 years, 89 ± 12 kg, 176 ± 8 cm) and nine normotensive controls (NT group, systolic blood pressure < 120 mmHg, diastolic blood pressure < 80 mmHg) matched for age, weight, and height (aged 40 ± 11 years, 80 ± 9 kg, 175 ± 5 cm). Participants were recruited through pamphlets and posters targeting untreated stage 1–2 hypertensive men in the local community. Screening included at least three daily automated blood pressure measurements (HEM-742INT; Omron Healthcare, Kyoto, Japan) over 2 days. Hypertension was confirmed using 24-h ambulatory blood pressure monitoring (Dyna-MAPA; Cardios, São Paulo, Brazil). Eligibility criteria excluded individuals who were engaged in regular exercise training, were undergoing pharmacological treatment, or had a history of smoking, cardiovascular events, or other chronic diseases. Additionally, participants exposed to high altitudes (≥ 2000 m above sea level) within the past 6 months were excluded.

### Experimental protocol

After providing written informed consent, participants were familiarized with the study protocol and instructed to abstain from food, caffeinated beverages, alcohol, and exercise for at least 24 h before the session. Participants returned for testing at least 48 h after the initial screening and familiarization. Upon arrival at the facility, participants were instrumented and rested in a supine position in a dark, quiet, temperature-controlled room (~ 24 °C). During a 20-min normoxic (NX) baseline phase, participants breathed a gas mixture containing 21% O_2_ and 79% N_2_ to establish a target eupneic PetCO_2_ level while breathing at a spontaneous rate (12–15 breaths per minute). Participants then performed a 5-min NX trial, breathing at a controlled rate of 20 respiratory incursions per minute using a metronome while maintaining the target PetCO_2_ level. This standardized breathing rate was based on our previous studies [15–17] and implemented to ensure consistent PetCO_2_ levels across conditions and to prevent ventilatory distress during subsequent hypoxia.

Following the NX trial, a 5-min isocapnic hypoxia (IH) trial was initiated using a gas mixture of 10% O_2_ and 90% N_2_ to achieve a target oxygen saturation range of 75–80%. During the IH trial, tidal volume was allowed to increase and was voluntarily modulated through verbal instruction to maintain the target PetCO_2_. IH was maintained using a partial rebreathing circuit that comprised a tee at the mouthpiece, in which one inlet provided a controlled supply of fresh or hypoxic gas, while the second inlet allowed re-inspiration of alveolar gas whenever minute ventilation exceeded the flow of fresh gas [19, 20]. This system enabled stable PetCO_2_ levels to be maintained, even under conditions of increased ventilatory volume [16, 17, 21].

### Measurements

Heart rate was continuously monitored using a lead II electrocardiogram (BioAmp, MLA2540; ADInstruments, Bella Vista, NSW, Australia). Beat-to-beat mean blood pressure (MBP) was measured via finger photoplethysmography (Finometer Pro; Finapres Medical Systems, Arnhem, Netherlands) and validated against right brachial artery pressure measurements (EM-759-E; Omron Healthcare). Breathing-related changes in abdominal or thoracic circumference were recorded using a piezoelectric transducer (MLT1132 respiratory belt; ADInstruments). Arterial oxygen saturation was measured through earlobe oximetry (Oximeter Pod; ADInstruments).

Participants breathed through either a mouthpiece (with a nose clip) or a mask connected to a gas analyzer (Ultima CPX; Medgraphics, St. Paul, MN, USA), enabling the measurement of breath-by-breath ventilation, tidal volume, and partial pressure of end-tidal oxygen (PetO_2_) and carbon dioxide (PetCO_2_). MSNA was recorded using microneurography [4, 22–29]. A unipolar tungsten needle electrode (FHC, Bowdoinham, ME, USA) was inserted into a muscle fascicle of the right peroneal nerve near the fibular head, with a reference electrode placed away from the recording site. Adjustments to the recording electrode were made to achieve a signal with the following characteristics: a pulse-synchronous pattern of spontaneous bursts with a signal-to-noise ratio of at least 3:1, no responses to unexpected loud noises or skin stroking, and a significant increase during an end-expiratory breath-hold or Valsalva maneuver.

The raw neurogram signal was processed by amplification (×100,000), filtration (bandwidth 700–2000 Hz), rectification, and integration (time constant 0.1 s) to produce the mean voltage neurogram (Iowa Bioengineering, Iowa City, IA, USA). Data were recorded at a sampling rate of 1000 Hz and stored for offline analysis using PowerLab 16/35 hardware and LabChart 8 software (ADInstruments). Normalization of the mean voltage neurogram was achieved by calibrating the height of the largest set of bursts during baseline to a value of 1000 arbitrary integration units (AU). MSNA was quantified as burst incidence (BI, bursts/100 heartbeats), burst frequency (BF, bursts/min), and total activity (BF multiplied by mean burst area, AU/min) by computing the mean values over a 5-min duration of each trial.

### Sympathetic transduction

Sympathetic transduction was assessed using a time-domain signal averaging technique [27, 31]. We used open-source software to track changes in MBP over 15 consecutive cardiac cycles following each MSNA burst [32]. Cardiac cycles were classified based on the presence or absence of a preceding MSNA burst, and MBP changes were subsequently averaged at each corresponding time point. Sympathetic transduction was defined as the peak of MBP changes (peak Δ MBP) observed across burst-associated cardiac cycles, while MBP response to non-burst cycles was quantified as the MBP nadir (nadir Δ MBP).

### Blood pressure variability (BPV)

BPV was assessed using indices including standard deviation (SD), range, interquartile range (IQR; the difference between the 25th and 75th percentile subjects), coefficient of variation (CV; calculated as [SD/mean] × 100), and average real variability (ARV) [12, 23, 33]. These indices were calculated for systolic (SBP), diastolic (DBP), and mean (MBP) blood pressure during both NX and IH.

### Heart rate variability (HRV)

HRV was quantified following the guidelines of the Task Force of the European Society of Cardiology and the North American Society of Pacing and Electrophysiology [34]. The ECG data collected were first analyzed and screened to remove ectopic beats. A minimum 350-beat segment of stable ECG data without ectopic beats and artifact-contaminated cardiac cycles was selected for the analysis. Time-domain HRV was obtained via the square root of the mean of the sum of successive differences in R-R intervals (RMSSD). Spectral analysis of HRV employed a fast Fourier transformation, and the power spectra were quantified by calculating the area under the following frequency bands: very-low-frequency (VLF) power (< 0.04 Hz), low-frequency (LF) power (0.04–0.15 Hz), and high-frequency (HF) power (0.15–0.4 Hz). Normalized units were computed by dividing each spectral band by the total power minus the VLF power, multiplied by 100. The LF to HF power ratio (LF/HF) was also calculated as an index of cardiac autonomic balance.

### Spontaneous sympathetic baroreflex sensitivity (sBRS)

sBRS was quantified using the method described by Kienbaum et al. [35] with the sympathetic neural activity analysis toolkit in Ensemble (Elucimed, Wellington, New Zealand). To account for the conduction delay of sympathetic outflow to the common peroneal nerve, the MSNA nerve tracing for each participant was shifted by approximately 1.2–1.4 s. Diastolic blood pressure (DBP) values were grouped into 3-mmHg bins to minimize non-baroreflex influences such as respiratory effects [23, 30]. For each bin, the corresponding MSNA burst incidence was calculated. sBRS was determined by plotting MSNA burst incidence against mean diastolic BP for each bin. Each data point was weighted based on the number of cardiac cycles, as bins at extreme diastolic pressure contained fewer cycles [23, 30]. The slope of the weighted linear regression (acceptance level: *r* ≥ −0.7) provided the spontaneous sBRS for each participant [36].

### Spontaneous cardiac baroreflex sensitivity (cBRS)

cBRS was assessed using the sequence technique, which identifies progressive increases (up sequences, cBRSup) or decreases (down sequences, cBRSdown) in systolic BP (SBP, ≥ 1 mmHg), followed by corresponding lengthening or shortening of the R-R interval (≥ 1.0 ms) over three or more consecutive heartbeats (CardioSeries v2.4) [23]. Linear regression analysis was applied to determine the slope of the relationship between SBP and R-R intervals for all identified sequences, with a minimum acceptance threshold of *R*^2^ ≥ 0.85. Separate analyses were performed for up sequences (cBRSup), down sequences (cBRSdown), and the total sequences (cBRSall), and the results are reported accordingly [23].

### Statistical analyses

All variables were continuously recorded and averaged over a 5-min period. Data normality was assessed using the Shapiro–Wilk test. Comparisons between HT and normotensive NT groups were performed using unpaired Student's *t*-tests for continuous variables and chi-square tests for categorical variables. Primary outcomes, including MSNA, sympathetic transduction, BPV, sBRS, and cBRS were analyzed using a two-way mixed-model analysis of variance (ANOVA). Multiple comparisons were conducted with two-tailed paired or unpaired Student's *t*-tests, as appropriate. Statistical analyses were performed using IBM SPSS (version 26) and RStudio (version 1.4.1103). We also conducted a stepwise linear regression that included sBRS, cBRS, MSNA BF, and sympathetic transduction (peak Δ MBP and nadir Δ MBP) as likely predictors of MBP variability. Data are expressed as means ± SD unless otherwise noted, with statistical significance set at *p* ≤ 0.05.

## Results

As previously reported, baseline characteristics such as age, body mass, height, and body mass index (BMI) were similar between the experimental groups, except for systolic and diastolic blood pressure (Table 1), which were intentionally higher in the HT group by design. IH elicited comparable reductions in PetO_2_ and SaO_2_ alongside similar increases in HR and $(\dot{\text {V}}\text {E})$ across the groups. MBP, SBP, DBP, and PetCO_2_ remained unchanged during IH.

**Table 1:** Hemodynamic, respiratory, and neural responses to normoxia (21% O_2_) and isocapnic hypoxia (10% O_2_) in normotensive and hypertensive men

	NT		HT		Hypoxia	Group	Interaction
	21% O_2_	10% O_2_	21% O_2_	10% O_2_			
Hemodynamics							
SBP (mmHg)	116 ± 7	117 ± 7	152 ± 11	152 ± 11	0.08	< 0.001	0.19
DBP (mmHg)	72 ± 6	73 ± 7	89 ± 11	86 ± 16	0.38	0.009	0.29
MBP (mmHg)	87 ± 6	87 ± 7	109 ± 8	107 ± 12	0.29	0.009	0.38
HR (bpm)	59 ± 8	81 ± 8	66 ± 11	80 ± 12	< 0.001	0.56	0.05
CO (L/min)	6 ± 1	8 ± 2	7 ± 2	9 ± 3	< 0.001	0.41	0.98
TC (L/min/mmHg)	0.7 ± 0.01	0.9 ± 0.01	0.6 ± 0.02	0.8 ± 0.03	< 0.001	0.31	0.24
Ventilatory							
${\dot{\text{V}}}$E (L/min)	11 ± 2	21 ± 5	15 ± 3	25 ± 11	< 0.001	0.21	0.90
PetO_2_ (mmHg)	106 ± 14	37 ± 5	105 ± 6	41 ± 5	< 0.001	0.38	0.45
PetCO_2_ (mmHg)	38 ± 3	38 ± 4	37 ± 3	37 ± 2	0.29	0.78	0.22
SaO_2_ (mmHg)	99 ± 1	81 ± 4	98 ± 1	82 ± 4	< 0.001	0.59	0.33
Neural							
BF (bursts/min)	12 ± 6	20 ± 6*	27 ± 12†	38 ± 95*^†^	< 0.001	0.04	0.03
BI (bursts/100 Hb)	20 ± 10	25 ± 9	44 ± 21	53 ± 20	< 0.001	0.06	0.17
Total activity (AU/min)	3242 ± 1604	3979 ± 1259	5355 ± 2568	6170 ± 2581	0.01	0.13	0.87

Mean ± SD

*BF* burst frequency, *BI* burst incidence, *DBP* diastolic blood pressure, *Hb* heartbeats, *HR* heart rate, *HT* hypertensive, *MBP* mean blood pressure, *NT* normotensive, *PetO*_*2*_ end-tidal oxygen partial pressure, *PetCO*_*2*_ end-tidal carbon dioxide partial pressure, *SaO*_*2*_ oxygen saturation, *SBP* systolic blood pressure, $\dot{V}$*E* minute ventilation

*p < 0.05 21% O_2_ vs. 10% O_2_

^†^*p* < 0.05 normotensive vs. hypertensive

MSNA was recorded in five subjects from each group. Under normoxic conditions, the HT group demonstrated a higher MSNA BF. Although BI was elevated in the HT group, the difference between groups did not reach statistical significance. A significant time–group interaction indicated that the HT group experienced a more pronounced increase in MSNA BF (NT: +8 ± 2 vs. HT: +12 ± 2 bursts/min, *p* = 0.039) during IH. A significant time effect indicated that BI (HT: +9 ± 3 bursts/100 heartbeats vs. NX, *p* < 0.001) and total activity only increased in the HT group in response to IH (HT: +2524 ± 2673 AU/min vs. NX, *p* = 0.050).

**Fig. 1:**
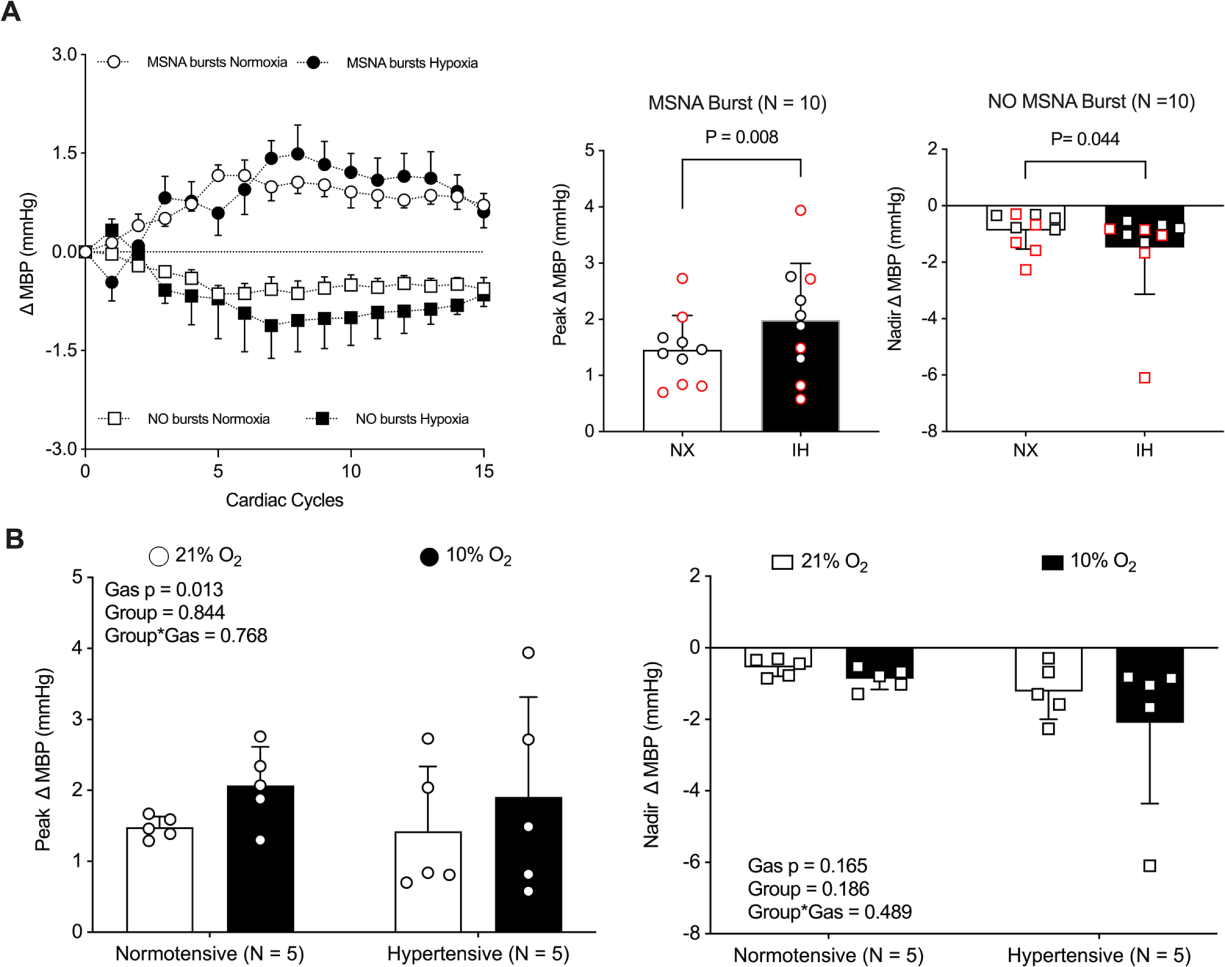
Average and peak changes (Δ) in mean blood pressure (MBP) across 15 cardiac cycles following spontaneous MSNA bursts and non-bursts are presented for pooled data (**A**) and for separate group analyses (**B**). For the pooled analysis (*N* = 10), a paired Student *t*-test was used, and the red circles and squares indicate hypertensive individuals. For the separate group analyses (*N* = 5 per group), a two-way mixed-model ANOVA was performed

Figure 1A illustrates beat-to-beat changes in mean blood pressure (Δ MBP) across 15 cardiac cycles following spontaneous MSNA bursts (top of the right panel) and non-bursts (bottom of the right panel). An exploratory paired *t*-test combining data from NT and HT participants revealed that IH significantly increased peak Δ MBP (Fig. 1A, middle panel; NX: 1.4 ± 0.6 mmHg vs. IH: 2.0 ± 1.0 mmHg, *p* = 0.008). When NT and HT groups were analyzed separately under NX, MBP increased similarly following a spontaneous burst (Fig. 1B, left panel). A significant main effect of time indicated that IH elicited a comparable increase in sympathetic transduction—reflected by a greater MBP response after spontaneous bursts—in both NT and HT individuals. When data were pooled, IH significantly reduced nadir Δ MBP during non-burst cardiac cycles (Fig. 1A, left panel; NX: −0.9 ± 0.6 mmHg vs. IH: −1.5 ± 1.6 mmHg, *p* = 0.044). However, when analyzed separately by group (Fig. 1B, right panel), neither hypertension nor hypoxia significantly altered the nadir Δ MBP observed during non-burst cycles.

**Fig. 2:**
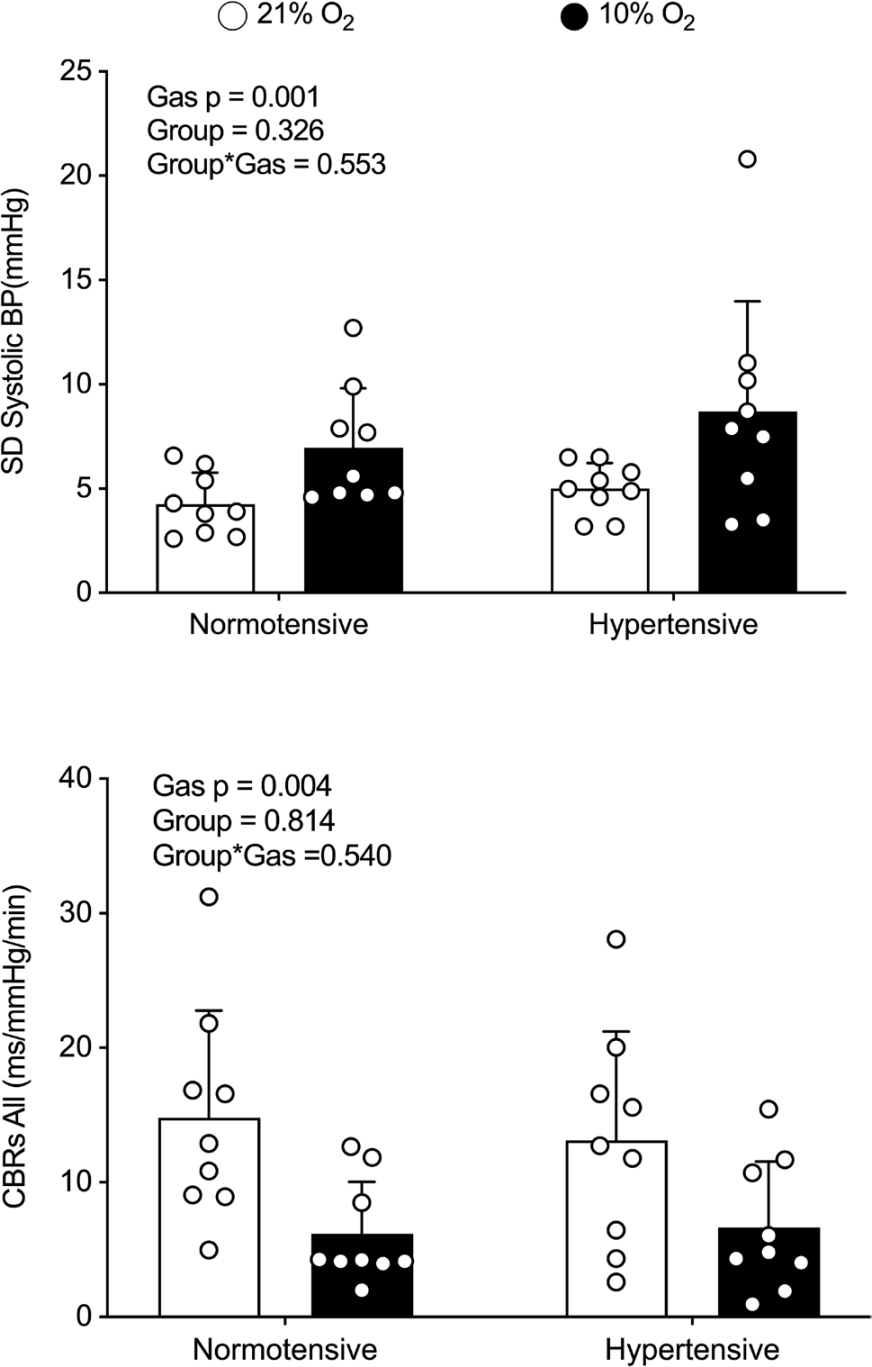
Systolic blood pressure standard deviation (SBP SD) and cardiac baroreflex (CBRS all) during normoxia (21% O_2_) and isocapnic hypoxia (10% O_2_) in normotensive and hypertensive men. Data were analyzed using a two-way mixed-model ANOVA with nine individuals per group

**Fig. 3:**
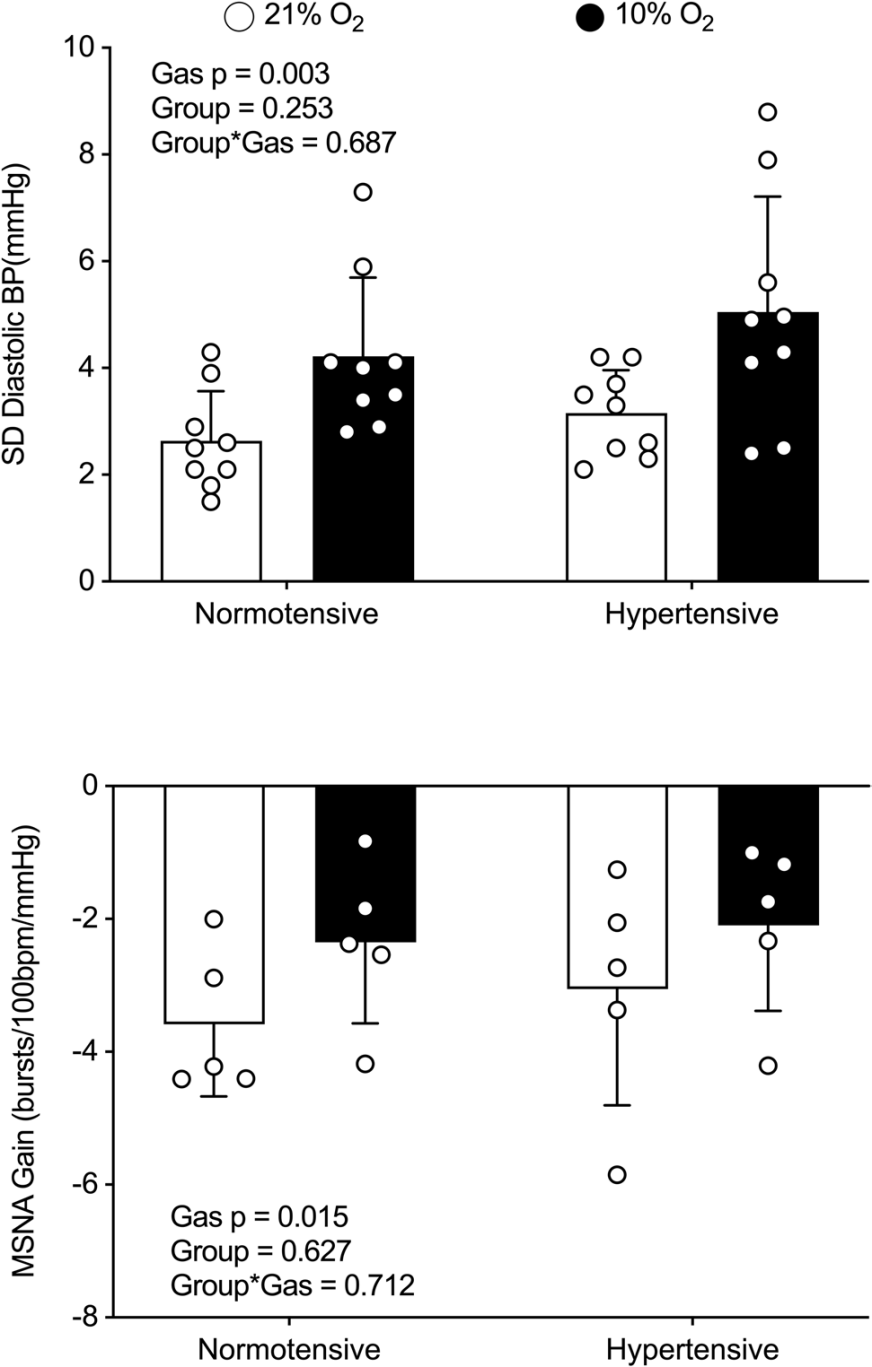
Diastolic blood pressure standard deviation (DBP SD) and sympathetic baroreflex (MSNA gain) during normoxia (21% O_2_) and isocapnic hypoxia (10% O_2_) in normotensive and hypertensive men. Data were analyzed using a two-way mixed-model ANOVA. DBP SD analyses included nine individuals per group, while MSNA gain analyses included five individuals per group

BPV, HRV, cBRS, and sBRS were similar between groups (Table 2) at NX. Exposure to IH led to comparable increases in SBP and DBP standard deviations across groups, accompanied by reductions in cBRSall (Fig. 2) and sBRS (Fig. 3). Other BPV measures also showed consistent increases in response to IH across both groups, while the remaining indices of cBRS decreased during desaturation. Notably, IH induced a comparable reduction in RMSSD, but no significant changes were observed in other HRV indices.

**Table 2:** Blood pressure and heart rate variability and cardiac baroreflex responses to normoxia (21% O_2_) and isocapnic hypoxia (10% O_2_) in normotensive and hypertensive men

	NT		HT		Hypoxia	Group	Interaction
	21% O_2_	10% O_2_	21% O_2_	10% O_2_			
BPV							
SD							
MBP (mmHg)	2.9 ± 1.2	4.9 ± 1.8	3.5 ± 0.9	5.9 ± 3.9	< 0.001	0.31	0.60
SBP (mmHg)	4.3 ± 1.6	7.0 ± 2.8	4.9 ± 1.3	8.4 ± 5.6	0.01	0.32	0.55
DBP (mmHg)	2.6 ± 1.0	4.2 ± 1.5	3.2 ± 0.8	5.0 ± 2.3	< 0.001	0.25	0.68
CV							
SBP (%)	3.7 ± 1.3	6.0 ± 2.7	3.3 ± 0.7	5.7 ± 3.6	0.01	0.74	0.91
DBP (%)	3.6 ± 1.4	6.0 ± 2.4	3.5 ± 0.8	6.0 ± 3.8	0.008	0.83	0.71
MBP (%)	3.4 ± 1.3	5.7 ± 2.4	3.1 ± 0.7	5.8 ± 3.4	< 0.001	0.95	0.79
ARV							
SBP (mmHg)	1.4 ± 0.4	1.7 ± 0.6	1.6 ± 0.4	2.0 ± 0.8	0.005	0.31	0.65
DBP (mmHg)	1.3 ± 0.5	1.3 ± 0.5	1.7 ± 0.4	1.7 ± 0.5	0.89	0.09	0.96
MBP (mmHg)	1.1 ± 0.4	1.3 ± 0.4	1.4 ± 0.3	1.7 ± 0.6	0.009	0.10	0.51
HR variability							
RMSSD	45 ± 23	27 ± 21	39 ± 22	26 ± 22	0.02	0.44	0.57
LF (nu)	48 ± 25	64 ± 20	51 ± 19	51 ± 19	0.15	0.56	0.30
HF (nu)	52 ± 25	41 ± 21	50 ± 20	50 ± 18	0.29	0.74	0.52
LF/HF (nu)	1.4 ± 1.0	2.4 ± 2.1	1.4 ± 1.1	1.3 ± 0.9	0.25	0.27	0.35
cBRS							
cBRSup (ms/mmHg)	15 ± 9	5 ± 3	14 ± 8	7 ± 5	< 0.001	0.90	0.44
cBRSdown (ms/mmHg)	14 ± 7	7 ± 4	12 ± 9	6 ± 5	0.001	0.60	0.66
cBRSall (ms/mmHg)	15 ± 8	6 ± 4	13 ± 8	7 ± 5	< 0.001	0.81	0.54

Mean ± SD

*ARV* average real variability, *BPV* blood pressure variability, *cBRS* cardiac baroreflex sensitivity, *CV* coefficient of variation, *DBP* diastolic blood pressure, *HF* high frequency, *HT* hypertensive, *HR* heart rate, *LF* low frequency, *MBP* mean blood pressure, *NT* normotensive, *RMSSD* square root of the mean of the sum of successive differences in R-R intervals, *SBP* systolic blood pressure, *SD* standard deviation

In the stepwise linear regression model, three independent variables—sBRS, MSNA BF, and sympathetic transduction—were retained as significant predictors of MBP variability. All three showed a positive and statistically significant association with MBP SD (*p* < 0.05), with MSNA BF exhibiting the strongest standardized effect (*β* = 0.440; *p* = 0.01), followed by sBRS (*β* = 0.430; *p* = 0.01) and peak Δ MBP (*β* = 0.361; *p* = 0.03), respectively.

## Discussion

Hypertensive individuals exhibit heightened carotid body sensitivity and amplified sympathetic activation in response to acute hypoxia [4, 5]. This exaggerated sympathetic response has been linked to paradoxical vasoconstriction, contrary to the expected vasodilation, in certain vascular networks [4]. Despite this, acute hypoxia does not elicit significant hypertensive responses in these individuals [4, 5], suggesting a potential impairment in sympathetic transduction under hypoxic conditions. While acute blood pressure responses may not fully capture the increased cardiovascular risk posed by hypoxia in hypertensive individuals, emerging evidence suggests that blood pressure variability (BPV)—the beat-to-beat fluctuations in blood pressure—may be a more accurate predictor of cardiovascular risk and end-organ damage. Elevated BPV is strongly associated with heightened sympathetic nerve activity [13] and transduction [37] and reduced arterial baroreflex sensitivity [38]. We hypothesized that HT individuals would exhibit more significant BPV during hypoxia than their normotensive counterparts, driven by exaggerated sympathoexcitation and more pronounced impairments in sympathetic transduction and baroreflex sensitivity. Contrary to our expectations, although the HT group exhibited exaggerated sympathetic responses to hypoxia, the increase in BPV was comparable to that observed in NT peers. This similar BPV response was accompanied by parallel increases in sympathetic transduction and comparable impairments in cBRS and sBRS across groups, underscoring the complex interplay between hypoxia, baroreflex function, and cardiovascular regulation in hypertension.

Although the HT group exhibited elevated resting BP and MSNA, sympathetic transduction and spontaneous cardiac and sympathetic baroreflex sensitivity were comparable to those of their normotensive counterparts. These findings challenge previous reports suggesting impaired sympathetic transduction [9] and reduced arterial baroreflex sensitivity in hypertensive individuals [37, 38]. In our study, sympathetic transduction appeared to be preserved in the HT group, as MBP increased similarly following an MSNA burst in both groups. Furthermore, we extended the analysis to demonstrate that MBP also decreased to a similar extent during sequences of cardiac cycles in which no MSNA bursts occurred. While sympathetic transduction appeared preserved in HT individuals based on time-domain signal averaging, the overall pattern still implies that higher MSNA levels may be required to elicit vasoconstrictor responses equivalent to those observed in NT individuals. This interpretation aligns with the concept that in the context of chronically elevated sympathetic activity, the vasculature becomes less responsive to adrenergic stimulation—possibly due to desensitization or reduced expression of adrenergic receptors, structural vascular remodeling, and/or compensatory mechanisms aimed at limiting excessive vasoconstriction. Factors such as age, the absence of pharmacological therapy, and the underlying etiology of hypertension may explain preserved sympathetic transduction and baroreflex function observed in our HT group [39]. While the small sample size (*n* = 5 per group) limits the ability to fully assess the impact of hypertension on sympathetic baroreflex function, additional findings such as the higher MSNA BF, preserved cBRS regardless of the direction of blood pressure fluctuations, and the lack of significant differences in HR and BP variability further support the similarities between the two cohorts in our study.

In line with previous studies [4, 5], IH elicited a significantly greater sympathetic response in the HT group than in their normotensive counterparts. Given the nature of the hypoxic stimulus—IH—this heightened response is likely attributable to the increased carotid chemoreceptor sensitivity associated with hypertension [4, 5], despite similar ventilatory responses between groups. Although the comparable increase in ventilation does not directly support the enhanced carotid chemoreceptor sensitivity, it is important to note that, by design, the respiratory rate was fixed at 20 breaths per minute. As such, the hyperventilatory response was exclusively driven by increases in tidal volume, which participants voluntarily modulated to maintain the target PetCO_2_ during IH. Despite exhibiting elevated MSNA, as indicated by BF, the HT group did not display a hypertensive response to IH. We initially hypothesized that the dissociation between heightened sympathetic activation and the absence of a pressor response would be explained by impaired vascular transduction. However, contrary to this hypothesis and aligned with previous evidence [8], sympathetic transduction increased during IH—not only when data from both groups were pooled but also when NT and HT individuals were analyzed separately—as indicated by greater MBP changes following MSNA bursts in both groups. Interestingly, when data from both NT and HT groups were pooled, IH elicited a more significant reduction in MBP during cardiac cycles without MSNA bursts. Although no significant changes were detected when NT and HT were analyzed separately, the magnitude of the IH-induced MBP reduction during non-burst cycles was comparable to the MBP increase observed following MSNA bursts in both groups. As such, these offsetting changes likely account for the absence of significant alterations in overall MBP and DBP during IH despite the exaggerated increase in MSNA.

Unlike absolute blood pressure, BPV increased similarly in response to IH in both NT and HT individuals, regardless of the representative indices used. In both groups, SBP SD, CV, and ARV consistently increased. This was accompanied by a significant positive chronotropic response, increased CO, and impaired cBRS, irrespective of the direction of BP fluctuations. A similar trend was observed for DBP, where increases in SD, CV, and ARV were accompanied not only by changes in sympathetic transduction but also by an impaired sBRS. Although our study design did not permit direct identification of the mechanisms by which hypoxia impairs baroreflex sensitivity, carotid vasodilation [39] and a subsequent reduction in the transduction of blood pressure into barosensory vessel stretch may explain these findings. Additionally, alterations in central processing and sympathetic transduction induced by hypoxia could also play a significant role in modulating baroreflex sensitivity and cannot be excluded as potential contributors to our observations.

Our findings diverge from the original hypothesis that greater sympathetic activation—along with more pronounced disturbances in transduction and baroreflex sensitivity—in response to IH would lead to higher BPV, specifically in hypertensive individuals. However, results from the stepwise linear regression model suggest that, in partial alignment with our hypothesis, alterations in sympathetic activity, transduction, and arterial baroreflex sensitivity independently contribute to elevated mean BPV during IH, irrespective of hypertensive status. This interpretation is consistent with prior evidence linking increased BPV to heightened MSNA [12, 13], reduced baroreflex sensitivity [38], and enhanced sympathetic transduction [37]. Unlike BPV, HRV indices were not consistently altered by IH. The positive chronotropic response to IH appeared to be mediated by parasympathetic withdrawal, as RMSSD showed a significant reduction in both HT and NT. While changes align with the observed chronotropic responses exhibited by the study groups under IH, other HRV indices, such as LF, HF, and LF/HF ratio, did not change significantly. This lack of consistent alteration in these indices suggests that changes in cardiac autonomic outflow may not fully explain the mechanisms underlying the observed HR responses.

### Limitations

As noted earlier, the study's sample size, particularly for MSNA analysis, was relatively small, which represents a limitation. Nevertheless, we believe that this did not compromise the ability to thoroughly evaluate the effects of hypertension on sympathetic transduction and sympathetic baroreflex function. Additional observations support this conclusion, including the higher MSNA BF, the preserved cBRS irrespective of the direction of BP fluctuations, and the absence of significant differences in HR and BPV. These findings collectively reinforce the similarities between the two cohorts in our study. A notable limitation of this study is the absence of female participants, which restricts the generalizability of our findings to only male populations. Sex differences in cardiovascular and autonomic regulation are well documented, with women typically exhibiting lower sympathetic nerve activity, higher parasympathetic tone, and different baroreflex responses compared to men. Including women in future studies is essential to better understand how these physiological differences may affect the mechanisms we investigated and to provide a more comprehensive perspective on the interplay between sex, autonomic function, and hypoxia. Although none of the participants had a clinical diagnosis of obstructive sleep apnea (OSA), we did not conduct formal screening to rule out undiagnosed cases. Given the high prevalence of undetected OSA in hypertensive individuals and its known impact on sympathetic activity and chemoreflex sensitivity, this also represents a limitation of the present study.

## Conclusion

 Untreated hypertensive individuals exhibited increases in BPV comparable to their normotensive peers during acute IH, despite demonstrating exaggerated sympathetic activation in response to oxygen desaturation. This elevation in BPV was accompanied by a similarly increased sympathetic transduction and impairments in both cardiac and sympathetic baroreflex control across normotensive and hypertensive groups.

## Data Availability

The datasets generated and analyzed during the current study are available from the corresponding author upon reasonable request.
